# Anandamide, Acting via *CB2* Receptors, Alleviates LPS-Induced Neuroinflammation in Rat Primary Microglial Cultures

**DOI:** 10.1155/2015/130639

**Published:** 2015-05-18

**Authors:** Natalia Malek, Katarzyna Popiolek-Barczyk, Joanna Mika, Barbara Przewlocka, Katarzyna Starowicz

**Affiliations:** ^1^Laboratory of Pain Pathophysiology, Institute of Pharmacology, Polish Academy of Sciences, Smetna 12 Street, 31-343 Krakow, Poland; ^2^Department of Pain Pharmacology, Institute of Pharmacology, Polish Academy of Sciences, Smetna 12 Street, 31-343 Krakow, Poland

## Abstract

Microglial activation is a polarized process divided into potentially neuroprotective phenotype M2 and neurotoxic phenotype M1, predominant during chronic neuroinflammation. Endocannabinoid system provides an attractive target to control the balance between microglial phenotypes. Anandamide as an immune modulator in the central nervous system acts via not only cannabinoid receptors (CB1 and CB2) but also other targets (e.g., GPR18/GPR55). We studied the effect of anandamide on lipopolysaccharide-induced changes in rat primary microglial cultures. Microglial activation was assessed based on nitric oxide (NO) production. Analysis of mRNA was conducted for M1 and M2 phenotype markers possibly affected by the treatment. Our results showed that lipopolysaccharide-induced NO release in microglia was significantly attenuated, with concomitant downregulation of M1 phenotypic markers, after pretreatment with anandamide. This effect was not sensitive to CB1 or GPR18/GPR55 antagonism. Administration of CB2 antagonist partially abolished the effects of anandamide on microglia. Interestingly, administration of a GPR18/GPR55 antagonist by itself suppressed NO release. In summary, we showed that the endocannabinoid system plays a crucial role in the management of neuroinflammation by dampening the activation of an M1 phenotype. This effect was primarily controlled by the CB2 receptor, although functional cross talk with GPR18/GPR55 may occur.

## 1. Introduction

Neurodegenerative disorders, such as Alzheimer's disease, Parkinson's disease, multiple sclerosis, stroke, and chronic pain, are associated with ongoing inflammation in the central nervous system (CNS) [1–6]. One of the striking hallmarks of these neurodegenerative disorders is chronic microglial activation. Microglial cells are of monocytic origin and play the role of the resident phagocytes of the innate immune system in the CNS. However, there is a lack of information about the initial signals that trigger microglial activation; neuronal depolarization, extracellular ion changes, nitric oxide (NO), and proinflammatory cytokines may contribute to microglial reactivity [7–9]. In our study, the activation of microglia was induced by lipopolysaccharide (LPS), a widely described agonist of toll-like receptor-4 (TLR-4) that is responsible for stimulation of the immune system [10].

Recent studies have shown that activated microglia can be divided into two phenotypic profiles. The classical M1 state, characterized by proinflammatory factors for example, interleukins (IL-1*Β*, IL-18, and IL-6) and inducible nitric oxide synthase (NOS2) [11–14], is neurotoxic and therefore contributes to secondary neuronal damage, cell death, and demyelination, which lead to neurodegeneration [15, 16]. The neuroprotective M2 state, known as “alternative activation,” is associated with the release of anti-inflammatory factors, such as IL-10, IL-4, and NGF [13, 17, 18]. There is a paucity of information about the regulation of microglial polarization. Several studies indicate that the endocannabinoid system provides an attractive target for managing microglial-derived neuroinflammation and may regulate many aspects of the brain's inflammatory response, including the release of M1 phenotype specific cytokines [19]. The endocannabinoid system modulates both neuronal and immune functions through two protein-coupled cannabinoid receptors (CB1 and CB2), although endocannabinoids, especially anandamide (AEA), can activate numerous other receptors like PPARS, TRPV1, and GPR18/GPR55 [20]. The latter, involved in immunological responses, represents an interesting molecular target for the control of neuroinflammation [21–23]. Both cannabinoid receptors are expressed in microglia and may act as immune modulators in the CNS [24, 25]. Moreover, it has been proposed that microglia are the main population of cells responsible for the production of AEA in the CNS, as primary microglial cultures produce approximately 20-fold more of this compound than neuronal or astrocyte cultures [26]. Therefore, it is important to investigate the effect of AEA on microglia, especially during activation, as studies by Liu et al. [27] have shown increased production of AEA in macrophages after LPS induction.

Many of the neurodegenerative conditions of the CNS result in increased levels of endogenous AEA [28–30]; therefore, the endocannabinoid system may provide an attractive target to influence microglial phenotype during chronic inflammation. Therefore, to increase our understanding of the role of the endocannabinoid system in the modulation of microglial polarization, we explored the possible therapeutic action of AEA and AM-251, AM-630, and CID-16020046 at cannabinoid and GPR18/GPR55 receptors in the* in vitro* model of LPS-induced microglial activation.

## 2. Materials and Methods

### 2.1. Microglial Cell Cultures and Treatments

Primary cultures of microglial cells were prepared from 1-day-old Wistar rat pups as previously described [31, 32]. Briefly, cells were isolated from the rats' cerebral cortices and were plated at a density of 3 × 10^5^ cells/cm^2^ in a culture medium that consisted of DMEM/GlutaMAX/high glucose (Gibco, USA), supplemented with heat-inactivated 10% fetal bovine serum (Gibco, USA), 100 U/mL penicillin, and 0.1 mg/mL streptomycin (Gibco, USA). Cells were placed on poly-L-lysine-coated 75-cm^2^ culture flasks and were maintained at 37°C and 5% CO_2_. The culture medium was changed after 4 days. The loosely adherent microglial cells were recovered after 9 days by mild shaking and centrifugation. Microglial cells were suspended in a culture medium and plated at a final density of 2 × 10^5^ cells onto 24-well plates and 4 × 10^4^ cells onto 96-well plates. Adherent cells were incubated for 48 h in culture medium before being used for the analyses. Cell specificity was determined using an antibody to OX-42 (a microglial marker) in cultures of primary microglia. Levels of* C1q* (a microglial marker) and* Gfap* (an astroglial marker) mRNA were also investigated. Cultured primary microglia were more than 97% positive for OX-42 and* C1q*. Primary microglial cell cultures were treated with compounds (Table 1) for 15 min each and then for 24 h with LPS (100 ng/mL) (lipopolysaccharide from* Escherichia coli* 0111:B4; Sigma-Aldrich, USA) for mRNA analysis. In the case of coadministration of AM-251, AM-630, and CID-16020046 with AEA, antagonists were administered 15 min before AEA.

### 2.2. Griess Assay

The Griess method was used to quantify aqueous nitrite concentrations. The Griess method involves a colorimetric measurement of the concentration of nitrite ions (NO_2_^−^), which are a stable, nonvolatile breakdown product of NO. Medium collected from above the tested cells (50 mL) was transferred to 96-well plates in triplicate. Griess A reagent was added (1% sulfanilamide in 5% phosphoric acid) to the medium, and after 10 min incubation at room temperature, Griess B reagent was added (0.1%, dihydrochloride N-(1-naphthyl)-ethylenediamine). The absorbance of samples was read at *λ* = 540 nm. The results are presented as a percentage of released nitric oxide relative to the positive control (cells treated with LPS).

### 2.3. LDH Assay

Compound cytotoxicity was measured using a Lactate Dehydrogenase (LDH) Cytotoxicity Detection Kit (Roche, Basel, Switzerland). LDH is a stable cytoplasmic enzyme present in all cells, which is released to the cell medium during plasma membrane damage. Medium from cells was collected 24 h after LPS stimulation and placed in 96-well plates. Tetrazolium salt was then added for 30 min. Sample absorbance was measured at a wavelength of *λ* = 492 nm using a spectrophotometer (LabSystems Multiskan, LabX, Canada). The results were expressed as a percentage of the absorbance in negative control cells (cells not treated with LPS or other compounds).

### 2.4. RNA Preparation and Quantitative Real-Time PCR

Cell samples were collected in Trizol Reagent (Invitrogen, Carlsbad, CA, USA) and homogenized by pipetting and vortexing. RNA was isolated according to Chomczynski's method [33]. The total RNA concentration was measured using a NanoDrop ND-1000 Spectrometer (Nano-Drop Technologies, Wilmington, DE, USA). Reverse transcription of total RNA (500 ng per sample) was performed using iScript reverse transcriptase (Bio-Rad, Hercules, CA, USA), according to the manufacturer's protocol. qPCR reactions were performed using Assay-on-Demand TaqMan probes and TaqMan Universal PCR Master Mix (Applied Biosystems, Foster, CA, USA), according to the manufacturer's protocol. The following probes were used: Rn01527840_m1 (*Hprt1*), Rn02758689_s1 (*Cb1*), Rn04342831_s1 (*Cb2*), Rn04244746_m1 (*C1q*), Rn00566603_m1 (*Gfap*), Rn00561420_m1 (*Il-6*), Rn01483828_m1 (*Cox2*), Rn00580432_m1 (*Il1b*), Rn00587615_m1 (*Il-13*), Rn01422083_m1 (*Il-18*), Rn01456866_m1 (*Il-4*), Rn00563409_m1 (*Il-10*), Rn03037213_s1 (*Gpr55*), Rn01493247_m1 (*Gpr18*), Rn01533872 (*Ngf*), Rn00561646_m1 (*Nos2*), and Rn01525859 (*Tnf*). Reactions were run on a Real-Time PCR iCycler IQ (Bio-Rad, Hercules, CA, USA) with version 3.0 of the software. Cycle threshold values (Ct) were calculated automatically. Expression of the* Hprt1* (hypoxanthine phosphoribosyltransferase 1) transcript was quantified to control for variation in cDNA amounts. The abundance of RNA was calculated as 2^(−normalized threshold cycle⁡)^.

### 2.5. Statistical Analyses

The experimental data were obtained for experimental groups as follows: *n* = 4–6 for the analysis of the expression of receptors and inflammatory factors at the mRNA level and *n* = 10–15 for the Griess and LDH biochemical tests. All data are presented as the mean ± S.E.M. The results are presented as a % of expressing cells for glial markers, as a fold-change relative to the negative control for mRNA expression and LDH results, and as a % of the negative control for the assay of released nitrogen oxide (Griess test). The results were statistically analyzed using GraphPad Prism 5 (Version 5.0.4, GraphPad Software), which was used to perform a one-way analysis of variance ANOVA and Bonferroni post hoc test on the results. Differences with *P* < 0.05 were considered statistically significant. Charts were prepared using GraphPad Prism v.5.04 (GraphPad Software, USA). The graphs indicate statistical significance according to the scheme: +, *P* < 0.05; ++, *P* < 0.01; +++, *P* < 0.001; and ++++, *P* < 0.0001, where ∗ indicates a significant difference compared to the nonstimulated control, # indicates a significant difference compared to the LPS-stimulated control, and $ indicates a significant difference compared to the LPS-stimulated cells treated with AEA.

## 3. Results

### 3.1. The Concentrations of Compounds Used in the Biochemical and Molecular Analyses Did Not Show Signs of Toxicity

**Figure 1:**
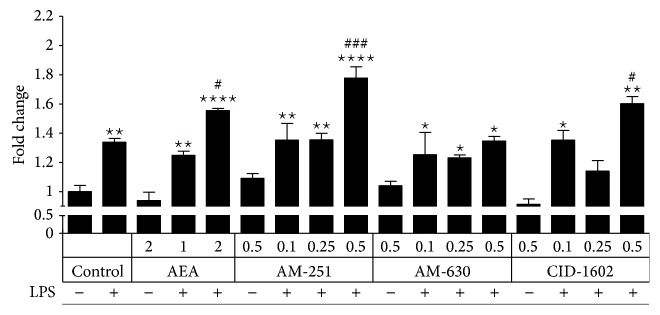
Compound cytotoxicity determined using the LDH assay. The test was performed 24 hours after LPS stimulation. The data are presented as the mean ± SEM and represent the normalized averages derived from 10 to 15 samples per group. The results are presented as fold-change relative to the unstimulated control. Statistical analysis was performed using one-way ANOVA followed by Bonferroni post hoc tests. Statistical *P* values <0.05 were considered significant. ∗ It denotes a significant difference versus the nonstimulated control; #, versus the LPS-stimulated control.

LPS treatment (100 ng/mL) resulted in an increase in LDH release in primary microglial cultures (Figure 1). Pretreatment with 2.0 *μ*M AEA resulted in additional cytotoxicity, which was not present after pretreatment with 1.0 *μ*M AEA, so this concentration was chosen for use in subsequent experiments. None of the concentrations of AM-630 tested showed cytotoxicity in the LDH assay when administered with LPS; thus, 0.5 *μ*M was chosen for the biochemical and molecular analyses. AM-251 and CID-16020046 resulted in increased release of LDH when administered with LPS at the highest concentration; therefore, 0.25 *μ*M, the highest nontoxic dose, was used for both compounds in subsequent experiments (Figure 1). None of the compounds used showed signs of cytotoxicity when administered without LPS (only the data for the highest dose is shown).

### 3.2. Involvement of Cannabinoid and GPR Receptors in the AEA-Mediated Alleviation of NO Production

**Figure 2:**
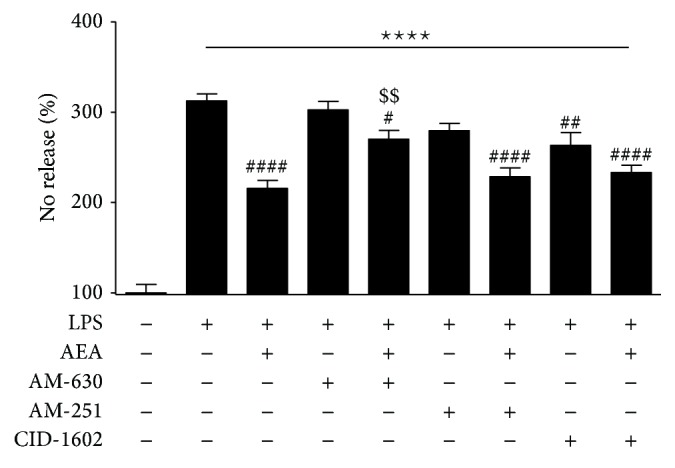
Release of nitric oxide in cultured rat primary microglia after LPS stimulation and pretreatment with AEA and AM-630, AM-251, or CID-16020046. The results are expressed as the percentage of NO release relative to the nonstimulated control (100%), 24 hours after LPS stimulation. Data are presented as the mean ± SEM and represent the normalized averages derived from 10 to 15 samples per group. Statistical analysis was performed using one-way ANOVA followed by Bonferroni post hoc tests. Statistical *P* values <0.05 were considered significant. ∗ It denotes a significant difference versus the nonstimulated control; #, versus the LPS-stimulated control; $, versus the LPS-stimulated cells treated with AEA.

An increase in the secretion of nitric oxide (NO) was observed (312.50 ± 7.81% of nonstimulated control, Figure 2) 24 h after the administration of LPS (100 ng/mL). AEA (1 *μ*M) administered 30 min prior to LPS stimulation reduced NO production by approximately 30% (215.55 ± 8.84% of nonstimulated control, Figure 2). To determine the involvement of the respective receptors in the AEA-mediated effect, coadministration with AM-630 (0.5 *μ*M), AM-251 (0.25 *μ*M), and CID-16020046 (0.25 *μ*M) was performed in LPS-stimulated cells. Pretreatment with AM-630 compensated for the effect of AEA by approximately 40% compared to NO release in AEA- and LPS-treated cells (270.14 ± 9.77% of nonstimulated control, Figure 2). Antagonism of CB1 or GPR18/GPR55 did not change the level of released NO in AEA- and LPS-treated cells. Treatment with AM-630 and AM-251 alone did not significantly diminish NO production compared to the LPS-stimulated control.

### 3.3. Alteration of* Cb1*,* Cb2*,* Gpr18*, and* Gpr55* Expression in LPS-Stimulated Primary Microglial Cells after Treatment with the Tested Compounds

**Figure 3:**
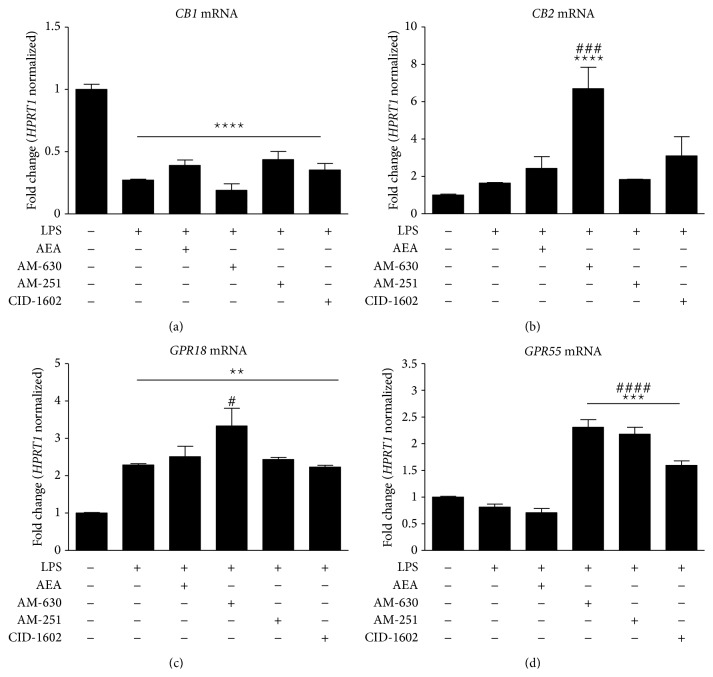
Expression of CB1, CB2, GPR55, and GPR18 transcripts in rat primary microglial cultures in the presence of the tested compounds. Samples were analyzed 24 hours after the stimulation of cells with LPS. Data are presented as the mean ± SEM and represent the normalized averages derived from 6 to 8 samples per group. The results are presented as the fold-change normalized to the expression of the reference gene* Hprt1* and were calculated relative to nonstimulated cells. Statistical analysis was performed using one-way ANOVA followed by Bonferroni post hoc tests; *P* values <0.05 were considered significant. ∗ It denotes a significant difference versus the nonstimulated control; #, versus the LPS-stimulated control.

CB1, CB2, GPR18, and GPR55 transcripts were detected in both nonstimulated and LPS-stimulated primary microglial cell cultures. LPS stimulation significantly decreased the level of CB1 mRNA regardless of the compound used for treatment (Figure 3(a)). Significant upregulation of* Cb2* expression was observed after AM-630 treatment in LPS-stimulated cells. Moreover, CB2 transcript levels tended to increase after AEA and CID16020046 treatment, although the changes were not significant (Figure 3(b)). LPS stimulation increased expression levels of* Gpr18* in all treatments, with the highest expression in AM-630-induced cells (Figure 3(c)). However, although no alteration of* Gpr55* in LPS-stimulated cells was observed, pretreatment with AM-630, AM-251, or CID-16020046 resulted in elevated expression of this receptor transcript (Figure 3(d)).

### 3.4. Expression of M1 Phenotype-Related Molecules in Rat Primary Microglial Cultures after Treatment with LPS and the Tested Compounds

**Figure 4:**
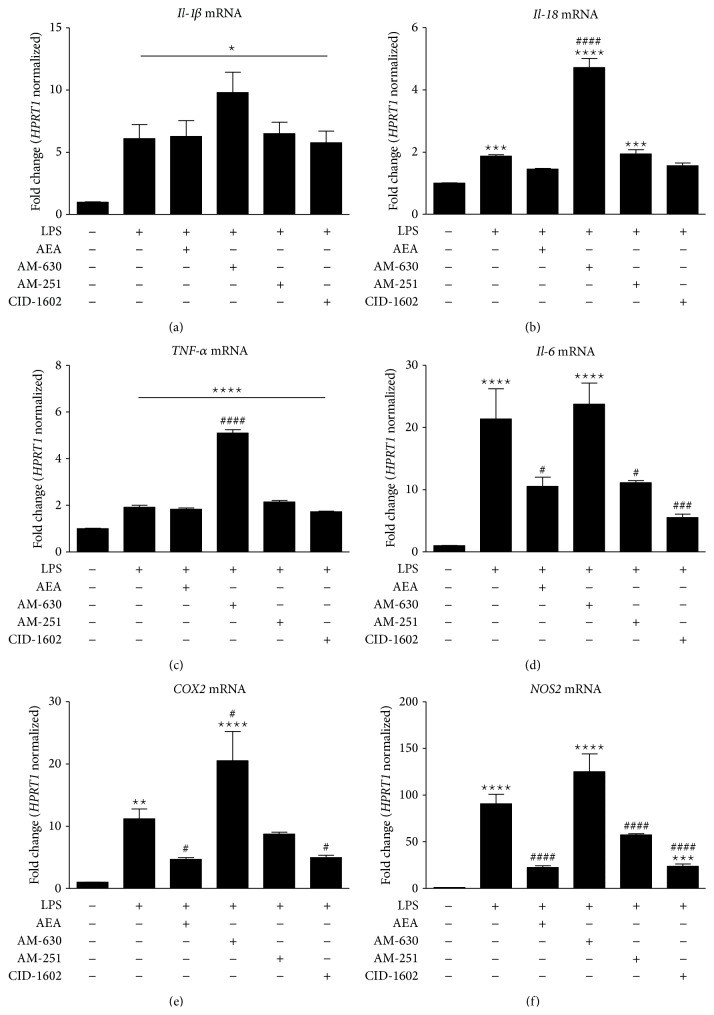
The results of qPCR analysis of the M1 phenotypic markers* Tnf-α*,* Il1β*,* Il18, Il6, Cox2, *and* Nos2 *gene expression in rat primary microglial cultures in the presence of the tested compounds. Samples were analyzed 24 hours after LPS stimulation. Data are presented as the mean ± SEM and represent the normalized averages derived from 6 to 8 samples per group. The results are presented as fold-change normalized to the expression of the reference gene* Hprt1* and were calculated relative to nonstimulated cells. Statistical analysis was performed using one-way ANOVA followed by Bonferroni post hoc tests; *P* values <0.05 were considered significant. ∗ It denotes a significant difference versus the nonstimulated control; #, versus the LPS-stimulated control.

All of the tested proinflammatory factors showed significant upregulation after LPS stimulation.* Il-1β* showed an upward trend after AM-630 administration, although the change was not statistically significant (Figure 4(a)).* Il-18* expression tended to decrease after AEA treatment, although the change was not significant. AM-630 treatment resulted in significant upregulation of* Il-18 *expression (Figure 4(b)).* Tnf-α* expression tended to increase after LPS stimulation, although the increase was significant only after AM-630 treatment (Figure 4(c)). Upregulation of IL-6 mRNA in LPS-stimulated cells was attenuated after AEA treatment. Administration of AM-251 showed similar effects on the decrease in IL-6 mRNA levels, and CID16020046 showed an even greater effect (Figure 4(d)). In contrast, AM-630 treatment produced no effect on the LPS-induced increase in IL-6 transcript. Both* Cox2* and* Nos2* showed patterns of mRNA expression similar to that of* Il-6* after treatment with the tested compounds in LPS-stimulated cell cultures (Figures 4(e) and 4(f)).* Il-6* showed lower expression levels in LPS-stimulated cultures treated with AEA compared to LPS-stimulation alone. After AM-630 administration, the expression level of* Il-6* returned to that of LPS-treated cells.

### 3.5. Changes in M2 Phenotype-Related Molecules in LPS-Stimulated Rat Primary Microglia after Treatment with the Tested Compounds

**Figure 5:**
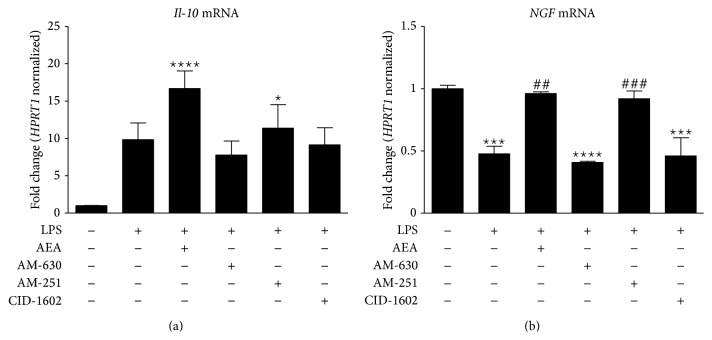
Expression of transcripts for the M2 phenotypic markers* Il-10* and* Ngf* in rat primary microglial cultures in the presence of the tested compounds. Samples were analyzed 24 hours after the stimulation of cells with LPS. Data are presented as the mean ± SEM and represent the normalized averages derived from 6 to 8 samples per group. The results are presented as fold-change normalized to the expression of the reference gene* Hprt1* and were calculated relative to nonstimulated cells. Statistical analysis was performed using one-way ANOVA followed by Bonferroni post hoc tests; values with *P* < 0.05 were considered significant. ∗ It denotes a significant difference versus the nonstimulated control; #, versus the LPS-stimulated control.

Expression of anti-inflammatory IL-10 mRNA was significantly elevated after AEA treatment in LPS-stimulated cells (Figure 5(a)). Moreover, administration of AM-251 also showed similar results. After LPS stimulation, the expression of* Ngf* was significantly decreased. AM-630 and CID-16020046 did not alter NGF transcript levels in LPS-stimulated cell cultures (Figure 5(b)). In contrast, after treatment with AEA or AM-251, levels of NGF mRNA returned to the level of the nonstimulated control. Expression of IL-4 and IL-13 mRNA was below the detection level in both nonstimulated and LPS-stimulated primary microglial cell cultures.

## 4. Discussion

In the present study, we have demonstrated that the alleviating effect of AEA on NO production in primary microglial cultures is mediated mainly through CB2 receptors. Activated upon CNS damage, microglia initiate and play a critical role in the development of CNS inflammation. Various stimuli can activate microglia, causing proinflammatory or anti-inflammatory functions depending on the duration, nature, and scale of the stimulus [34]. It has been shown that the inflammatory response of LPS-stimulated microglia, which leads to increased secretion of NO, contributes to events underlying brain inflammation and neuronal degeneration [35]. Biochemical studies performed on LPS-treated microglia have demonstrated similarity in the transcriptional activation of a large panel of inflammatory genes, including IL-1*β*, IL-6, and IL-18 [36], which are typical of the M1 phenotype of activated microglia.

Some studies have indicated that short-term cannabinoid exposure can have a neuroprotective effect at the time of the sudden failure of CNS tissues [37]. Bursts of AEA, which is synthesized “on demand” in areas of cellular stress (e.g., in damaged tissue or at the site of inflammation), have been suggested as the mechanism that inhibits the immune response in both normal and injured tissues, where it is involved in the migration of immune cells to the site of inflammation [38]. Our study demonstrated a reduction in NO release after pretreatment with AEA in LPS-stimulated primary microglial cultures, which suggests it has a neuroprotective action during CNS tissue damage. The decrease in NO production was correlated with a downregulation of* Nos2* expression, in the absence of AEA toxicity in the LDH assay. Thus, it can be concluded that the reduction of NO production was not due to the toxicity of the AEA but to inhibition of the inducible NO synthase. Although it has been previously suggested that CB1 is expressed constitutively [39], our study showed a decrease in the expression of the CB1 receptor upon microglial activation, which may prove their contribution in response to LPS treatment. Interestingly pretreatment with none of used compound influenced* Cb1* expression levels. However, it is difficult to clarify the involvement of CB1 in the inflammatory response, as both its agonists and antagonists demonstrate immunomodulatory functions [40].

We demonstrated that* Cb2* expression tended to increase after LPS stimulation, although only additional treatment with AM-630 elevated the CB2 transcript level significantly. This result supports the finding that* Cb2* expression is inducible upon inflammation [39]. Indeed, studies have shown that CB2 receptor activation reduces the immune response during CNS inflammation, brain edema, and the death of neurons, alleviating the symptoms of neurodegenerative diseases in animal models [41]. CB2 receptor stimulation inhibits the activation of microglia, slowing down the development of Alzheimer's disease [30]. Similarly, CB2 receptor activation in microglial cells in the spinal cord can reduce inflammatory reactions and pain after peripheral nerve injury [42–44]. Evidence of the modulation of CB2 expression after microglial activation is mixed. Some studies have shown that there is downregulation of CB2 receptor levels after activation [45], while others have reported that inflammatory stimuli upregulate CB2 microglial expression [46, 47].

Microglial activation is a polarized process that can be divided into M1 and M2 phenotypes [11, 48]. During the short-term activation of microglia, the presence of both the M1 and M2 phenotypes is balanced, allowing the restoration of CNS homeostasis; however, chronic inflammation causes a shift toward the proinflammatory M1 phenotype. One of the actions of activated microglia is the promotion of inflammation, which causes an influx of immune cells to the site of injury. To this end, the M1 phenotype of microglial cells initiates neuroinflammation by producing cytotoxic factors such as cytokines (e.g., IL-1*β*, IL-6, IL-18, and TNF-*α*) and enzymes (NOS2 and COX2), which, in addition to acting as chemoattractants, may lead to neuronal damage upon chronic activation [49]. In our previous work [13], we reported that LPS-treated cells are an important source of many proinflammatory (e.g., IL-1*β*, IL-6, and IL-18) and anti-inflammatory (e.g., IL-1*α* and IL-10) factors. Moreover, cannabinoids can modulate cytokine production [50], which in turn contributes to a reduction of the immune response and can be beneficial in autoimmune diseases. It has been shown that the high levels of AEA observed during CNS damage are responsible for its neuroprotective effects, affecting the TLR4-dependent activation of microglia [51]. Modulation of intracellular signal transduction pathways leads to changes in the expression of immune response-related genes.

IL-1*Β*, a marker of the microglial M1 phenotype, showed an elevated expression level after LPS treatment, which may suggest microglial polarization upon LPS activation. Similarly* Il-18* expression was also increased, which is consistent with studies in murine microglial cells [52]. Additional M1 phenotypic molecules,* Il-6 *and* Cox2*, showed upregulated expression in LPS-treated cells, which was alleviated by pretreatment with AEA, suggesting an anti-inflammatory action in LPS-stimulated primary microglial cultures. Moreover, decreased expression of* Cox2* may contribute to increased activation of the FAAH- and LOX-dependent metabolic pathways of AEA, which result in anti-inflammatory metabolites [53].* Tnf-α*, besides being upregulated upon LPS stimulation, showed elevated levels after AM-630 treatment. This result is consistent with studies showing suppression of* Tnf-α* expression associated with reduced NO production in microglial cells after treatment with the CB2 receptor agonist [46]. Moreover, it has been reported that stimulation of CB2 receptors causes a reduction in the release of proinflammatory cytokines, such as TNF-*α* and IL-6 [54].

During short-term activation of microglia, a balanced immune response is maintained by anti-inflammatory factors (e.g., IL-4, IL-13, IL-10, and NGF) produced by cells with the M2-phenotype, which allows the management of CNS inflammation [55]. In our studies,* Il-10* levels were significantly increased after AEA treatment. As IL-10 inhibits the release of IL-1*β* and IL-6, it may play an important role in the development of neuroinflammation [56]. NGF has a well-documented neuroprotective effect (for review see [57]) and is a specific marker of M2-phenotype microglia [12, 17]. Our study showed downregulation of* Ngf* expression after LPS stimulation, which was restored to baseline levels with AEA treatment.

We reported that AEA alleviation of NO production was not abolished by CB1 antagonism, although it was sensitive to CB2 antagonism. AM-630 partially blocked the AEA effect on NO production, together with the elevated expression of* Nos2* in LPS-induced primary microglial cultures. It has been shown that AEA inhibits NO release and* Nos2!* expression in an LPS-activated murine microglial cell line (BV2-cells). This effect is sensitive to CB2 but not CB1 antagonism, as indicated by the finding that neuronal death was even greater after AM-630, but not AM-251, administration [38]. Our findings, together with these results, strongly suggest that the endocannabinoid system, and in particular CB2 receptors, is involved in the regulation of neuroinflammation. Incomplete attenuation of the AEA effect by the CB2 antagonist may be due to the action of AEA on other molecular targets. CB1 and CB2 are well-documented targets of AEA, but there is also evidence of the activation of other receptors by this compound. Studies by Puffenbarger et al. [58] have demonstrated reduced levels of* Il-1β*,* Il-6,* and* Tnf-α* expression after cannabinoid administration in activated microglial cells, which is not sensitive to CB1 and CB2, suggesting the presence of an additional, as yet unidentified, cannabinoid receptor on microglia. Among the numerous candidates of particular interest are GPR18 and GPR55, which have been shown to be involved in the immunomodulatory effects, and the expression of which has been demonstrated on microglial cells [21, 59–61].

Interaction between GPR55 and CB2 in the control of inflammatory processes has been reported [22, 62, 63]. Although the expression of* Gpr55* was not elevated after LPS induction, pretreatment with AM-251, AM-630, or CID-16020046 resulted in a significant increase in GPR55 mRNA levels. These results support its hypothesized function in dampening excessive cannabinoid receptor activation [64]. Moreover, administration of AM-630 caused an increase in* Gpr18* expression, which may further suggest functional cross talk between CB2 and GPR18 receptors. Interestingly, recent findings have suggested that GPR18 may act as a new microglial target in the control of neuroimmunological episodes in the CNS (for review see [61]). Our data indicates* Gpr18 *expression is elevated after LPS induction, regardless of the compound used for treatment.

Although CID-16020046, a mixed antagonist of GPR18/GPR55, did not block the effect of AEA on NO production in primary microglial cultures in our studies, it decreased NO release when administered alone. The abolishment of NO production by antagonism of GPR18/GPR55 suggests that they may play a role as immune modulators. Because of the lack of selective pharmacological tools at this time, further studies using knockouts are needed to confirm this hypothesis.

Our studies showed that AEA causes a reduction in microglial cell activation, especially by dampening activation of the M1 phenotype. We demonstrated the involvement of the CB2 receptor in the cytoprotective effect of AEA. Moreover, we provided novel, interesting data of the involvement of GPR18/GPR55 in microglial activation. Summing up, the use of pharmacological tools to control the phenotype of microglia through the endocannabinoid system may be useful in the treatment of neurodegenerative conditions.

## Figures and Tables

**Table 1:** Compounds interacting with the endocannabinoid system used in the study.

Compound	Description	Used concentration	Vehicle	Producer, cat number
AEA	Endocannabinoid	1-2 *μ*M	10%EtOH	Tocris, #1339
AM-251	CB1 antagonist	0.1–0.5 *μ*M	2%DMSO	Tocris, #1117
AM-630	CB2 antagonist	0.1–0.5 *μ*M	2%DMSO	Tocris, #1120
CID-16020046	GPR18/55 antagonist	0.1–0.5 *μ*M	2%DMSO	Tocris, #4959

## Abbreviations

*AEA*: Anandamide
*AM-251*: CB1 receptor antagonist
*AM-630*: CB2 receptor antagonist
*CB1*: Cannabinoid receptor type 1
*CB2*: Cannabinoid receptor type 2
*CID-16020046*: GPR18/GPR55 receptors antagonist
*COX2*: Cyclooxygenase 2
*CNS*: Central nervous system
*FAAH*: Fatty acid amide hydrolase
*GPR18/GPR55*: G-coupled protein receptor 18/55
*HPRT1*: Hypoxanthine-guanine phosphoribosyltransferase 1
*IL-1β*: Interleukin-1*β*
*IL-4*: Interleukin-4
*IL-6*: Interleukin-6
*IL-10*: Interleukin-10
*IL-13*: Interleukin-13
*IL-18*: Interleukin-18
*LDH*: Lactate dehydrogenase
*LOX*: Lipoxygenase
*LPS*: Lipopolysaccharide
*NGF*: Nerve growth factor
*NO*: Nitric oxide (II)
*NOS2*: Nitric oxide synthase 2
*TLR-4:*: Toll-like receptor-4
*TNF*-*α*: Tumor necrosis factor-*α*
*TRPV1*: Transient receptor potential cation channel subfamily V member 1.
